# Development and evaluation of a nurse-led, tailored stroke self-management intervention

**DOI:** 10.1186/s12913-015-1021-y

**Published:** 2015-09-04

**Authors:** Lisa Kidd, Maggie Lawrence, Jo Booth, Anne Rowat, Sian Russell

**Affiliations:** Institute for Applied Health Research/School of Health & Life Sciences, Glasgow Caledonian University, Glasgow, UK; School of Nursing, Midwifery & Social Care, Sighthill Campus, Edinburgh Napier University, Edinburgh, UK

## Abstract

**Background:**

Community nurses are well placed to promote and support stroke survivors to engage in self-management. The aim of this study was to develop a stroke self-management intervention that could be tailored towards stroke survivors’ self-management needs, goals and levels of activation, in the first year post-stroke.

**Methods:**

Mixed method study, designed in accordance with the British Medical Research Council’s (MRC) guidance for the development and evaluation of complex interventions. The intervention was developed and evaluated in two phases. The intervention was underpinned by the theoretical concept of patient activation and was developed based on a review of published research on stroke self-management interventions and qualitative interviews and focus groups (phase 1). It was evaluated using qualitative interviews and focus groups with stroke survivors and stroke nurses (phase 2). Participants comprised 26 stroke survivors, between 3 and 12 months post stroke and 16 stroke nurses, from across three NHS Boards in Scotland.

**Results:**

The intervention consisted of a tailored self-management action plan, incorporating an individualised assessment of stroke survivor’s readiness to self-manage (using the Patient Activation Measure), goal setting and motivational interviewing. Evaluation showed that many of the individual components of the intervention were perceived as feasible and acceptable to both stroke survivors and stroke nurses.

**Conclusions:**

To our knowledge, this is the first UK study to explore the use of patient activation as a theoretical underpinning in stroke self-management research and to involve stroke survivors and stroke nurses in the design and development of a tailored, person-centred stroke self-management support intervention. The study findings provide the first step in understanding how to effectively develop and deliver stroke self-management support interventions to stroke survivors living at home in the first year following stroke. Further work is needed to develop and refine the intervention and identify how to effectively embed it into nurses’ routine clinical practice.

## Background

### Introduction

There is growing recognition that self-management support is effective for people affected by stroke. The physical and psychological impact of stroke, however, is a significant barrier to stroke survivors’ engagement in, and access to, self-management support services and interventions. Little is known about how best to develop interventions in a tailored way that address stroke survivors’ individualised needs and goals. There is also little current evidence of how to best support the implementation and integration of stroke self-management support interventions within clinical practice. The current study describes the development and evaluation of a nurse-led, person-centred, self-management support intervention which aimed to tailor the provision of support towards stroke survivors’ individualised needs, goals and levels of activation. The study also aimed to explore nurses’ perceptions of implementing and integrating stroke self-management support in clinical practice, in order to help inform the design, development and implementation of future interventions.

### Stroke

Stroke is the leading cause of global long-term adult disability [1]. It is estimated that there are up to 1.1 million stroke survivors living in the UK [2]. Over half of these have persistent stroke-related disability, with significant and complex physical, cognitive, and emotional deficits that require continued lifelong care and support to self-manage [2]. Stroke-related impairments can affect individuals’ health literacy skills; their abilities to seek out, interpret and act on complex health information, as well as creating challenges with mobility, transport, reading and writing, and confidence with social interaction [2, 3]. All of these issues are likely to hinder engagement in, and indeed exclude, a significant number of stroke survivors from, effective, timely and appropriate stroke self-management and self-management support [3, 4].

### Stroke and self-management support

There is a rich global policy context, which endorses self-management support as a critical component of long-term condition (LTC) management. Emerging evidence on self-management aligns with UK priorities in designing and developing services to specifically address stroke survivors’ self-management needs and facilitate health professionals, including nurses, to deliver appropriate, timely and personalised self-management support. This support can be conceptualised as the provision of educational and supportive interventions by service providers and the development of mechanisms which help health professionals to facilitate individuals’ self-management by helping to develop their personal skills and confidence related to managing, and making decisions about, their own health [5, 6].

The evidence base for self-management support is rapidly expanding. Established, generic self-management support ‘programmes’ include the Stanford Chronic Condition programme [7] in the United States, the Flinders programme [8] in Australia and the Expert Patient Programme in the UK [9]. In the UK, two stroke-specific programmes are currently being tested and rolled out. The *Bridges Self-Management Programme* [10] aims to train practitioners to help stroke survivors to develop self-management skills (http://www.bridges-stroke.org.uk) and *The Stroke Workbook* [11], which is a manual-based self-management programme developed in accordance with Leventhal’s Self-Regulatory Model [12]. However, these programmes focus on stroke survivors’ needs around the time of discharge and early transition back into the community, rather than their longer-term self-management needs, which often do not reveal themselves until individuals are discharged from acute, post-stroke services.

Despite the policy rich context surrounding self-management, a key challenge remains in how to design and develop self-management support interventions that align with individual’s self-management priorities and needs and how to most effectively embed them within the ‘real-world’ setting of professionals’ clinical practice [13, 14]. Recent evidence reveals that health professionals can be reluctant or fearful to change practice, lack support and flexibility in being able to assess and respond to individuals’ needs in a person-centred manner and at a strategic level, and there is a lack of frameworks, procedures and guidance to enable them to deliver tailored self-management support as an integrated part of routine practice [15–18]. Self-management support programmes also frequently fail because they do not focus on, and are not tailored towards, individual patients’ priorities and preferences, nor is there an assessment of their self-management needs or abilities [16], or the outcomes of self-management that are of importance to them such as improved recovery and physical symptoms, self-efficacy or using fewer health services [19]. Little, or no, assessment of self-management needs, abilities or personal priorities and outcomes occurs in clinical practice and yet an understanding of these would enable self-management support interventions to be more effectively tailored towards these and would help to embed and sustain self-management support interventions within the ‘real-world’ setting of clinical practice.

### Patient activation and tailoring of self-management support

The study described in this paper introduces the concept of ‘patient activation’ as a mechanism for assessing and understanding individuals’ self-management needs, abilities and priorities, how these shape their responses and behaviours towards engaging in self-management and how, informed by this information, health professionals can provide person-centred self-management support that is specifically tailored towards individuals’ personal self-management needs, abilities and priorities. ‘Patient activation’ is a behavioural concept [20] which is defined as the readiness and ability to take on the role of managing both decisions and behaviours related to health and healthcare [21], and lies at the core of self-management decision making and activity [22]. Individuals are said to have different ‘levels’ of activation which can provide a useful indicator of their current attitudes and beliefs towards self-management and the level of their needs and abilities and the types of support they may require form health professionals to engage more actively in self-management. Patient activation levels are viewed as a continuum from low (e.g. Level 1), where individuals tend to be passive and overwhelmed by the prospect of managing their own health and may not understand their role in the care process, through to high (e.g. Level 4), where individuals have adopted many of the behaviours needed to support their health but may be less able to maintain them in the face of illness or stressful life events [23]. It is believed that as patient activation level increases, so too does perceived control and a sense of empowerment to engage in self-management activity [20].

Drawing on evidence from the United States and emerging evidence from the UK, greater perceptions of activation have been found to be associated with greater success in self-management and health promotion as well as greater engagement in health-related decision making; whilst lower perceptions of activation have been found to be associated with barriers to engaging in self-management activity [20, 21, 24, 25]. It is now becoming accepted that a ‘one size fits all’ approach to supporting self-management is ineffective in engaging *all* individuals in self-management. Drawing further on the emerging evidence around patient activation [20], rather than only focussing on patient activation as an *outcome* of self-management, it can be used as a *tool*; a tool which can help health professionals to assess their patients’ readiness towards engaging in self-management and help guide them on the type and amount of self-management support that each individual is likely to need and respond to. The use of the concept of ‘patient activation’, as measured using the Patient Activation Measure (PAM) (described more in Table 1), developed by Judith Hibbard and colleagues [23] in this way is relatively new in the UK [20]. To our knowledge, the current study is the first in the UK to explore its use in underpinning stroke self-management research. It is also the first to involve stroke survivors and stroke nurses in the design and development of a tailored, person-centred stroke self-management support intervention, comprising individualised, systematic assessment of stroke survivors’ needs, expectations and abilities to self-manage and aligning nurses’ provision of support towards these. This paper describes the development and evaluation of the intervention, conducted in accordance with the British Medical Research Council’s (MRC) guidance for the development and evaluation of complex interventions [26, 27].

**Table 1:** Summary of the development of the Intervention

Activity	Aims	Methods/Sources/Analysis
Systematic literature review	i) To identify the feasibility, acceptability, and effectiveness of self-management interventions for community-dwelling stroke survivors to inform the development of the intervention in this study	• Published literature sourced via key health-related databases (including Cinahl, Medline, Embase, PsycInfo & Cochrane Controlled Trials Register), published in English language, between 2000 and 2012, using terms related to *‘self-management’* and *‘stroke’* as keywords and subject headings
		• Relevant studies meeting the following criteria were included: quantitative or qualitative evaluation of an intervention, focussed on community-dwelling stroke survivors aged 18 years and over, and where the intervention was labelled as ‘self-management’ or clearly underpinned by the principles of a ‘person-centred’ approach to self-management (as developed by Lawrence and Kinns, 2012)
	ii) To identify gaps in existing research on stroke self-management interventions	
		• Data extracted from relevant studies and quality appraisal conducted (by SR, LK, ML, JB, AR)
		• Findings synthesised and presented narratively
Qualitative semi-structured interviews & focus groups	i) To understand stroke survivors’ perceptions towards self-management	• Qualitative semi-structured interviews with *20 stroke survivors* & qualitative focus groups/telephone interviews with *11 stroke nurses* from three Scottish health boards (conducted by SR & LK).
		• All interviews/focus groups lasted approximately one hour.
	ii) To understand stroke nurses’ perceptions towards stroke self-management and stroke self-management support	• Interviews with *stroke survivors* were conducted in participants’ homes and specifically explored their perceptions and attitudes towards self-management and self-management support, in particular their needs, abilities and preferences.
		• Focus groups with *stroke nurses* were conducted in their practice base and specifically explored their perceptions and attitudes towards self-management, their current provision of tailored stroke self-management support and the context of, and barriers and facilitators to, its implementation in practice (including training and support needs). The interviews also aimed to discuss their views on what should be incorporated into the stroke self-management intervention that could be delivered within the context of their daily clinical practice and their specific training needs for delivering this. •Interviews/focus groups were audio-recorded,
		• Transcribed and thematically analysed to identify key issues and themes within the data (Braun and Clarke, 2006).
		• Emerging codes and themes were consistently questioned and reviewed by members of the research team (LK, SR, ML, JB, AR) and the advisory group to ensure credibility, transparency and trustworthiness of the emerging findings.
Completion of the Patient Activation Measure	i) To obtain a quantifiable measurement of patient activation	• Completion of the Patient Activation Measure (PAM),^2^ with *20 stroke survivors*, which was designed to provide a quantifiable measurement of how able and confident each individual felt to engage in their self-management (conducted by SR)
	ii) To determine ‘level of activation’ across the study sample	• The Patient Activation Measure is a patient-reported outcome measure (PROM) containing a series of 13 statements designed to assess the extent to which an individual feels that they have the responsibility, confidence and knowledge to self-manage (Hibbard and Gilburt, 2014). Individuals are asked to rate the degree to which they agree or disagree with each statement; responses are combined to provide a single score of between 0 and 100, which represents the individual’s perception of themselves as an active self-manager (higher scores = greater perceptions of activation) (Hibbard and Gilburt, 2014).
	iii) To ‘test out’ the PAM and identify any preliminary issues in its use (prior to evaluation phase)	
		• PAM data were analysed using descriptive statistics (AR)
		• During the interviews, stroke survivors were also asked for their thoughts on the content and wording of the questions in the PAM and its ease of use.

### Complex interventions and the MRC Framework

The MRC guidance conceptualises complex interventions as those which comprise multiple components that interact and involve behaviours (either in those delivering or receiving the intervention) with the purpose of changing one or more outcomes [26, 27]. Given this definition, self-management support interventions, such as the one described in this paper - with their level of complexity in both process and outcome - fall into the ‘complex interventions’ category. The most recent version of the British Medical Research Council’s (MRC) guidance for the development and evaluation of complex interventions [27] (from hereon in referred to as the MRC framework), incorporates greater attention to the use of qualitative methods and process evaluation methods, and a more nuanced appreciation of social, political and geographical contexts in which the development, evaluation and embedding of interventions are grounded within. Adhering to such a framework is essential as it enables researchers to give a more comprehensive description of their intervention - in particular their content, delivery and contexts in which they are implemented and evaluated - enabling more successful transfer into the wider knowledge base and practice setting [28]. Subsequently, this paper has been written in accordance with the MRC framework [27] and the TIDieR (Template for Intervention Description and Replication) checklist [28].

## Methods

### Study aim and design

The overall aims of the study were; i) to design and develop a prototype nurse-led, stroke self-management support intervention (referred to hereon in as ‘development phase’) and, ii) to pilot test the prototype intervention and qualitatively evaluate its feasibility and acceptability from the perspectives of stroke survivors and stroke nurses (referred to hereon in as ‘evaluation phase’).

### Ethical approval

Ethical approval for the study was received from the West of Scotland Research Ethics Committee and management approval from the NHS Research Scotland Permissions Coordinating Centre.

### Participants and recruitment

For both the *development* and *evaluation* phases, stroke survivor participants were identified from stroke nursing team caseloads and were recruited via an initial advertisement sent out by stroke nursing teams. Willing and eligible stroke survivors were recruited by the research assistant who explained the study to them in further detail and obtained informed consent (SR). Participants meeting the following criteria were eligible: i) diagnosis of stroke (defined as “a focal (or at times global) neurological impairment of sudden onset, and lasting more than 24 h (or leading to death), and of presumed vascular origin” by the World Health Organisation); ii) diagnosis of ischaemic or haemorrhagic stroke (except for sub-arachnoid haemorrhage); iii) discharged from hospital within the previous 12 months following ‘first’ stroke (defined as “first in a lifetime, people who have never had a stroke before” by the World Health Organisation); iv) living at home within one of the three NHS boards participating in the study; v) have a basic understanding of the English language and, vi) able to give signed informed consent (personally or by advocacy of a carer/family member).

In the *development phase,* twenty stroke survivors were recruited; eight from NHS Lanarkshire, six from NHS Fife and six from NHS Highland. Participants ranged in age from 43 to 84 years (mean 64 years). Participants included both males (*n* = 12, 60 %) and females (*n* = 8, 40 %) and were between 1–6 months post-stroke (*n* = 12, 60 %) or 7–12 months post-stroke (*n* = 8, 40 %). In total, six participants (30 %) reported one or more additional comorbidities, 14 (70 %) suffered from fatigue and 12 (60 %) had cognitive impairments. Half the sample (50 %) reported that they had moderate to moderately severe disability/symptoms (0–3) based on the Modified Rankin Scale (MRS).

In the *evaluation phase,* six stroke survivors (not previously involved in the development phase) were recruited (all from NHS Lanarkshire only) by stroke nurses. Participants included both males (*n* = 4) and females (*n* = 2) and were between 1–6 months post-stroke (*n* = 5) and one man was between 6 and 12 months post stroke. Five participants reported one or more comorbidities. Three participants reported slight disability/symptoms based on the MRS, and three reported moderate to moderately severe disability/symptoms.

Stroke nurse participants working within stroke nursing teams in NHS Lanarkshire, Fife and Highland were invited to participate. The stroke nurses in these teams were specialist, community based practitioners whose roles were to visit stroke survivors at home or in the community following discharge to support the transition between acute and primary care and address the longer-term needs of stroke survivors post-discharge. The three NHS Boards were broadly representative of the 15 NHS Boards that exist across Scotland, and encompassed areas of affluence and deprivation, and both urban and rural conurbations. No exclusion criteria in terms of grade, previous experience or length of time qualified was applied to the stroke nurse participants. Willing participants were recruited by the research lead (LK) who obtained informed consent. In the *development phase*, 11 nurses were recruited. Demographic data were not collected on the nurses, other than date qualified (range from 10–37 years) and length of time in post (range from 2 months to 13 years). In the *evaluation phase,* five nurses participated in the focus groups/telephone interviews (all from NHS Lanarkshire). Demographic data were not collected from the nurses, other than date qualified (range from 10–27 years) and length of time in post (range from 2 months to 10 years).

### Development phase methods & procedure

The purpose of this phase was to develop a ‘prototype intervention’, informed by a review of published literature on stroke self-management interventions and to offer an understanding of stroke survivors’ and stroke nurses’ perceptions of self-management and stroke self-management support in order to help inform the content, structure and delivery of the intervention developed in this study. This phase also aimed to gather data on stroke survivors’ perceived levels of activation, and in doing so, enabled the research team to ‘test out’ the choice of assessment questionnaire to be used in the intervention, the PAM (which provides a measure of ‘patient activation’ or readiness to engage in self-management) [23]. The methods employed in the development of the intervention have been described in Table 1.

### Evaluation phase methods & procedure

The purpose of this phase was to pilot test the ‘prototype’ intervention and qualitatively evaluate its feasibility and acceptability from the perspectives of stroke survivors and stroke nurses.

#### The Intervention

**Fig. 1:**
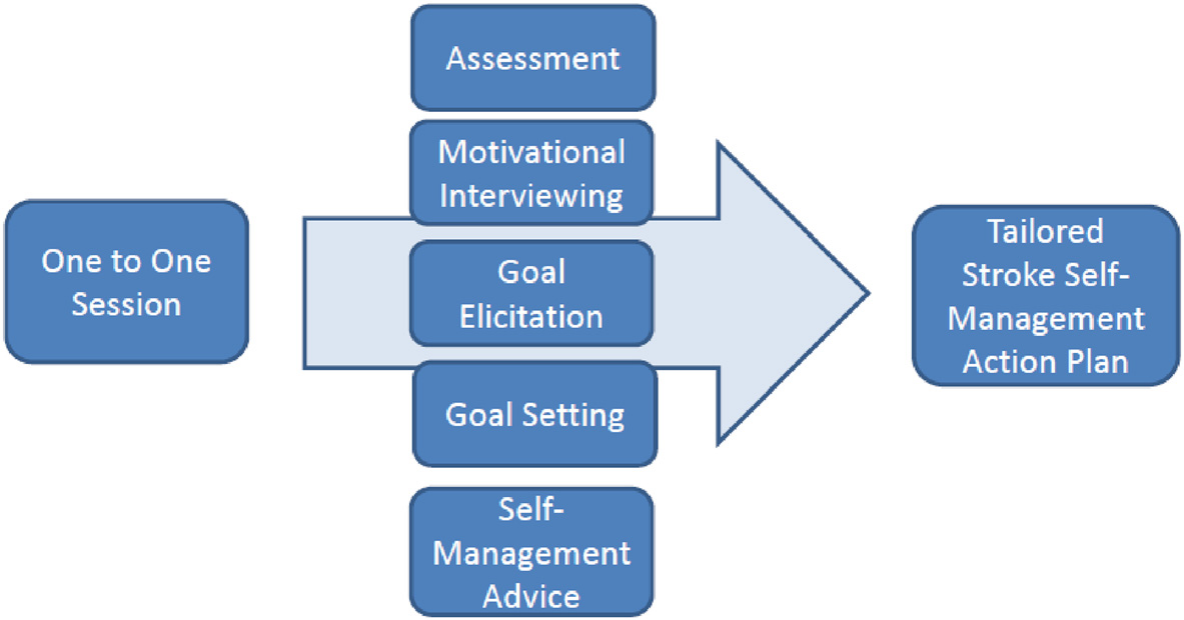
The components of the Intervention

The intervention took the form of a ‘tailored self-management action plan’, designed in a booklet format (included as supplementary material), and created by nurses and stroke survivors working in partnership using a structured self-management assessment questionnaire (The PAM) and a process of goal-setting. Figure 1 depicts the intervention elements and processes. As described in the sections that follow, goal setting was identified in the *development* phase as a key self-management support strategy within the review of stroke self-management interventions whilst the qualitative findings not only complemented this, but developed our nuanced understanding about the nature of the ‘goals’ set and the process itself. Motivational interviewing, appeared to be a complementary mechanism for encouraging the goal setting process to occur and was often featured in existing self-management interventions [29]. It is an approach that is guided by the principle of the client (stroke survivor), rather than the counsellor (nurse), evoking and voicing their motivations and arguments for change [30] and was considered a key feature of the intervention developed for the current study. Being mindful not to unduly increase participating nurses’ current workloads and to help determine general usability and relevance within the home practice setting, the intervention was purposely designed to be delivered by nurses within the contexts of their usual pattern of scheduled visits.

#### Procedure, training and fidelity

The intervention was delivered, by stroke nurses in one NHS Board (Lanarkshire), over a four-week period. The intervention was delivered face-to-face to individual stroke survivors in their homes in three separate sessions. The first session (week one) involved the ‘trial procedures’ such as information giving and completing the appropriate consent forms. The second session (week two) involved the completion of the PAM and the personally tailored self-management action plan. Responses to the PAM were documented and then used to frame a discussion around the identification of key self-management goals, using motivational interviewing prompts. Nurses then suggested specific self-management advice tailored to the context of individuals’ personal perceptions, abilities and confidence, as suggested within the appropriate activation-stage content of the tailored self-management action plan booklet. The third session (week four) involved reflection on the use of the plan, again using motivational interviewing prompts, and any necessary revisions to the on-going self-management plan.

The five nurses involved in the delivery of the intervention were trained by LK and SR. The training incorporated an educational session on the components of the intervention (e.g. goal setting, motivational interviewing, use of the PAM), reading materials and a copy of the study protocol, which outlined relevant study details including purpose and nature of the intervention, sample inclusion/exclusion criteria and intervention procedures, a list of motivational interviewing prompts that could be used during the intervention, face to face training in using the intervention, including patient vignettes, and role play to test out the intervention with volunteer stroke survivors who were specifically invited to take part in the training session through one of the clinical leads (not involved in the development or evaluation of the intervention itself). The training session was viewed positively, particularly the hands-on practice of delivering the intervention with volunteer stroke survivors. To help ensure fidelity, on-going monitoring, supervision, mentoring and debriefing was provided to the nurses via email and telephone contact during the intervention period (LK).

#### Evaluation questionnaires and focus group

At the end of the four-week intervention period, an open-ended evaluation questionnaire was completed by participating stroke survivors. The five nurses who delivered the intervention participated in a focus group (conducted by LK) which aimed to explore their views of delivering the intervention in practice and issues related to its design, delivery, future refinement, and its implementation and embedding within clinical practice. Descriptive data from the stroke survivors’ evaluation questionnaires were summarised. Focus group data were audio-recorded, transcribed and thematically analysed to identify key issues and themes within the data [31]. The emerging codes and themes were consistently questioned and reviewed by members of the research team (LK, ML, JB, AR) and the advisory group to ensure credibility, transparency and trustworthiness of the emerging findings. PAM score data were not collected in this phase as an outcome of the evaluation.

## Results

The findings from the development and evaluation phases are presented in the following sections. Verbatim quotations have been used to illustrate key themes generated from the qualitative data and these are presented in Tables 2, 3, 4, 5, 6 and 7. Abbreviations have been used to refer to participants as follows; SS to mean ‘stroke survivor’ and SN to mean ‘stroke nurse’, followed by a unique number. The words ‘Fife’, ‘Highland’ and ‘Lanarkshire’ following each abbreviation refers to the NHS Board in which participants lived/worked. In the evaluation phase, the stroke nurses’ comments from the evaluation focus group have been used as exemplars. These are presented in the form of a segment of narrative between the stroke nurses (denoted by SN, followed by a unique number) and with the interviewer (LK), rather than individual verbatim quotations. NHS Board is not stated since all participants lived/worked in NHS Lanarkshire.

**Table 2:** Stroke survivors’ perceptions of self-management

“It was a shock for me, and I was very scared about never being able to dance and exercise. So I worked very hard on myself, you know, even when I got home and I could only manage fifteen minute walk but, you know, I set myself little goals. My goal is, you know, to work towards my regaining, you know, my total fitness again for example I mean I am not unfit just now but…I want to try and get that back again.” (SS9, Highland)	
“I set myself goals, certainly at the beginning of things I was going to do and I’ve managed to do that so I think and now I’ve got the goal, if you like, of going back to work and I mean I think ‘oh I’m going to have to do it soon’, so I am organising that at the moment and that’s my next thing.” (SS14, Fife)	
“They [health professionals] don’t really want to know about you as an individual they just really want to dish out medicines and …erm… you know, they are all about risk rather than about helping someone to self-manage and live healthily with whatever has happened to them. My needs weren’t listened to. There isn’t really any facility really to pay attention to someone who wants to take responsibility for their own health, you know, there is no encouragement really to be able to do that.” (SS9, Fife)	
“The stroke nurse is very good and the physiotherapist, they always leave their phone number “phone me if ever you want to speak to me about anything” well okay that is very good but you never do it, or seldom do it. It’s only when push comes to shove that you do it.” (SS10, Lanarkshire)	
“I don’t think I’ve done anything off my own back. I always tell people like the physios I tell them what and I say “now is this okay or not okay” and they have been very keen for me to do this [exercising].” (SS10, Lanarkshire)	

*(SS denotes ‘stroke survivor’)

**Table 3:** Stroke nurses’ perceptions pre-intervention

“What hinders us is the lack of resources and services that are in Highland, and how remote our caseload is …erm… you’ve got some people that live in Inverness that have got access to services everywhere, which is fantastic, and then you’ve got patients fifty miles away that can’t access anything …and that is a severe barrier to being able to … get these people to self-manage, it’s just because they are on their own, you know, [and] unless they have got familyit’s very difficult.” (SN1, Highland)	
“There’s new websites, the new workbook, the lifestyle [course] you’ve [speaking to another nurse] been doing it a few years but it’s still a new concept and so it’s, what do you offer people? You know, somebody said “there is people that you don’t offer anything to if it’s appropriate” *[sic]* they are not at that stage or are never going to want that type of thing or there are people that are maybe at different stages and not maybe a group person and nobody wants to work through a manual and not everybody can go online. my view about some of the interventions as well, none of them are golden goose eggs in the sense that they help people in their recovery well some help some people …erm… but there is nothing that’s like some sort of magic wand.” (SN4, Lanarkshire)	
“I think that’s the challenges … we are always trying to find better ways to do things and [the question is] how that’s all joined up because, you know … there is always [*sic*] people coming up with different ways and ideas to look at things, and how does that become… joined up …?” (SN4, Fife)	
Intervention	
“Depending on what else you are doing that visit … you know, if you were going into this individuals’ to do that then it probably is okay … but if you had anything else to go over on that visit then no, it probably is a bit long … and to try and keep a stroke patients concentration for that long is very difficult, because I find myself, after about thirty minutes, you have lost them … (SN1, Highland)	

*(SN denotes ‘stroke nurse’)

**Table 4:** Stroke survivors’ perceptions post intervention

Q: Did you find the questions easy to understand?	
“Yes but people who have suffered worse than me might prefer more specific questions.” (SS1)	
“The questions were in plain English and straightforward.” (SS3)	
“The questions were easy to understand and appeared to be structured in such a way that answering them was simplistic. All you had to do was be honest and express your true feelings. [The questions] made you more aware of how your condition was impacting on everyday life.” (SS5)	
Q: How helpful did you find having the opportunity to identify and discuss your own goals/things that were important to you?	
“Very helpful…helps me to move forward in achieving my goal.” (SS2).	
“Very important to have something to aim for after a stroke to help with your recovery” (SS4).	
“It was helpful to discuss goals with my nurse. By working together I was able to suggest goals and we both decided if my suggestions were relevant to my condition, and most importantly, achievable. Small steps still get you to your destination.” (SS5)	
Q: How helpful did you find the specific self-management advice that was offered?	
“It was very helpful. It helped me to focus on the areas that were most important to me instead of trying to address all issues at the same time. If these areas can be improved, everything else could hopefully improve.” (SS5)	
“It was helpful as I was unsure of certain things.” (SS6)	
Q: Did you feel the advice you were offered was relevant and appropriate to your needs?	
“Yes very much so…we discussed in great detail where I wanted to be and how best to achieve this. It was a great help to speak to someone who had a high level of understanding of the problems I was facing and help to work out solutions.” (SS5)	
“Very relevant as I needed a lot of advice.” (SS6).	
Q: Do you feel you’ve learnt anything you didn’t know about your condition or managing things in your l ife that you didn’t know before?	
“I’ve learnt a lot and I am more able to understand things more.” (SS1)	
“Felt the people I talked to were very helpful and reassuring.” (SS3)	
“Definitely. [Stroke nurse] spent time explaining how stroke was impacting on my life i.e. the side effects I was experiencing. She also gave me more confidence by praising how well I was coping with the side effects.” (SS5)	
Q: Have you done anything differently to manage things in your life?	
“I’ve looked more into managing things by myself in my life.” (SS1)	
“I am eating better now after having problems and better after advice. I am no wiser about my condition though.” (SS4)	
“Having a better understanding allows me to now voice concerns which previously I didn’t as I thought the problems I was having were par for the course.” (SS5)	
“I now have definitely more focus on the important things to help me improve. There is also a more structured system in place for my daily activities as now I have a plan to follow. I praise myself more for achieving my own goals and I can now adjust these goals realistically. (SS5).	
Q: Do you feel more confident to manage things in your life than you felt before?	
“Yes…because of various agencies. I feel more confident to manage my condition.” (SS3)	
“No as I still need assistance at all times…I am no wiser about my condition.” (SS4)	
“Yes. I no longer allow the small setbacks to affect me as much. Previously my expectations were too high and I could feel dismayed if I did not meet them. I now have realistic goals which can be adjusted to suit my progress.” (SS5).	

*('SS' denotes 'stroke survivor')

**Table 5:** Documenting and recording goal setting

V1:	But probably you would have gone through the same process but just recorded it in your notes whereas you’re recording it in a goal format …
*LK:*	How different was it?
V1:	Hugely different [all voice agreement].
V4:	I suppose we *are* doing goal setting just now anyway we are just doing it *differently*. We are not doing the recording stage of it so how do you know what, I mean our patients still have goals; they say “this is what I want to do” and we are going to help them through that process. We are just not formally writing it.
V1:	And recording it.
V3:	… the girl I have done it with, she wouldn’t have verbalised that without me asking ‘what’s that goal that you want to be achieving?’ I was asking her about the forms, and she said…”It gives you a focus to know that’s there, and I’ve filled this in [the goal setting sections]

*V denotes the voice of each stroke nurse in the focus group

**Table 6:** Issues with PAM

V2:	Well I had a man like that that was questioning all that “I don’t know what you mean by that and I don’t know what you mean by that” [referring to individual PAM questions].
V3:	Whereas my lady was just “oh uh huh, yeah, aye, oh I agree with that, oh aye I agree with that”.
V1:	And it’s not something that the majority of people are familiar doing when it’s that type of thing, you know, the kind of questionnaire things are usually yes, no or whereas it was a bit more.
V3:	Aye so strongly agree was 4 and she’s got 1, 2, 3, 4, 5, 6, 7, 8, 9 [counting up the PAM question responses] ‘4’s and then the others [referring to other PAM question responses] are ‘3’s.
*LK:*	Yeah so that would kind of put her at the top end of the scale…
V3:	Yeah, [the scores indicate] she’s definitely gonna do that [self-manage].
*LK:*	You mean that it suggests she’s a very active self-manager and you wouldn’t say that she was?
V3:	She’s not doing anything.
V4:	Yep there was 1 or 2 he [referring to her patient] said that the language he didn’t feel was appropriate he felt that it was some of the words he knew what they meant but he was like that “this is really difficult”.
V4:	[my man] was thinking of other people and he was like that “I don’t think a lot of your patients with communication problems [would be able to understand the questions].”

*V denotes the voice of each stroke nurse in the focus group

**Table 7:** Challenges with implementation in practice

V4:	I can’t say I have been trying it with absolutely everybody …erm… I’ve had quite a difficult run of the types of patients I have had…there has been bigger issues [than self-management] I suppose that had to get dealt with.
*LK*:	*Yeah so it’s kind of been a kind of difficult caseload you’ve had?*
V3:	And it’s absolutely time.
V1:	And it’s adding onto your…[workload].
V3:	And that’s not what we would normally do.
V1:	And it’s trebling the workload.
*LK*:	*Yep so you think in terms of kind of workload it would put extra pressure on you?*
V3:	See but the goal setting part I don’t think is unachievable I think it’s more if you’ve to fill in all these other things afterwards [the action plan and PAM].

*V denotes the voice of each stroke nurse in the focus group

### Development phase

#### Review findings

The synthesised evidence demonstrated that self-management interventions (SMIs) for stroke survivors can have a positive impact on: quality of life, psychological and cognitive functioning and self-management behaviours, cognition and knowledge. There was scant evidence regarding the feasibility, appropriateness and meaningfulness of SMIs and interventions (components and their delivery) were often poorly described. Poor reporting of interventions is a well acknowledged issue in this field, and has clear implications for the development of evidence-informed interventions, as in the current study. However, goal setting, aided by a process of motivational interviewing, was consistently highlighted as a key self-management strategy, and was incorporated as a central component of our intervention.

#### Patient Activation Measure (PAM) Scores

Participants’ scores on the PAM were high (*n* = 20, median 75.3, IQ range 69, 80) (possible range 0–100). The majority of participants (*n* = 17, 85 %) were actively self-managing and were able to maintain this in times of stress, illness or anxiety (characteristic of level 4). Of the three participants who had lower PAM scores, two indicated that they had understanding of self-management, but the lacked confidence to actively engage (characteristic of level 3) and one indicated a lack of confidence and knowledge to self-manage (characteristic of level 2). All three participants suffered from fatigue and had moderate/severe disability (MRS ≥2).

Interestingly, although the majority of participants (*n* = 17, 85 %) reported high scores on the PAM, the individual narratives from the interviews often suggested that individuals did not necessarily perceive themselves as having responsibility for, and confidence and knowledge to engage in, their self-management, particularly in times of stress, illness or anxiety . This finding highlights that specific needs are perhaps not being addressed, and would be missed if nursing input was guided only by the total PAM score/activation scale.

In relation to the content of the PAM and its ease of use, six participants reported difficulty in answering three specific PAM statements. This may suggest that the tool is not sufficiently patient-centred [32] or stroke-specific. Particularly challenging statements included Question 8 (“*I understand my health problems and what causes them”).* Participants voiced that they might respond differently to the two distinct issues contained within that particular statement, in the context of stroke i.e. understanding what a stroke is and understanding the aetiology of their stroke.

#### Qualitative findings

The analysis of the qualitative semi-structured interviews (stroke survivors) and focus groups (stroke nurses) supported the literature review findings and both groups of participants identified ‘goal-setting’ as a fundamental component of stroke self-management. The qualitative findings suggested that it was the meaningfulness and perceived value of the ‘goals’ identified and articulated by individuals themselves that acted as a key mechanism for understanding attitudes towards self-management and helped to offer a framework through which nurses could support individuals to become actively engaged in self-management. In particular, participants who appeared to be the most actively engaged self-managers (i.e. those who spoke about personally engaging in a process of reflection and self-management decision making), were those who appeared easily able to identify and articulate personally meaningful goals. These goals appeared to form an established part of their mind-set and subsequent recovery and included getting back to work or being fit enough to re-engage in a specific hobby or interest. They were also able to clearly formulate a plan(s) to address and achieve such goals. Conversely, participants who spoke of a more nebulous desire to ‘get better’ and who were less able to easily identify and articulate specific goals appeared to self-manage in a more passive style e.g. they did not engage in the process of reflection and self-management decision making, but rather they followed and adhered *only* to the advice of medical and allied health professionals. They appeared fearful of engaging in any ‘new’ self-management activity that had not been’approved’ by their nurse. Nurses were viewed as having a pivotal role in supporting stroke survivors’ self-management; however, it was perceived that the support offered needed to focus on offering reassurance and security and building individuals’ confidence, involve more face-to-face interaction, and be more structured, focussing on individual’s own goals and wishes (see Table 2).

Stroke nurses perceived that they supported stroke self-management by educating patients and signposting them to relevant resources and information. They perceived goal setting as an important strategy but that individuals had to be ready, prepared and confident to accept their part in the process, and to take some responsibility for self-managing. The stroke nurses’ narratives revealed that the demands of the culture in which they were working did not support them in promoting and supporting engagement in self-management. They were particularly mindful that any additional self-management work that they undertook and facilitated should not add burden to their already demanding workload. They recognised the importance of their self-management role, however, they often struggled to individualise self-management support due to a lack of structured assessment processes and systems, the ability to tailor self-management support, and a lack of confidence in doing so. The issue of terminology arose during the focus group discussions; in particular, what terms would be used to refer to the intervention documentation. The terms inventory, guideline, and workbook were all discounted; however, the nurses appeared happy and had familiarity with the concept of a ‘tailored self-management action plan’ (see Table 3).

### Evaluation phase

#### Stroke survivors’ perceptions of the intervention

The sections of the ‘tailored self-management plan’ booklet were completed well by all participants and all reported that they were happy with the presentation and layout of the booklets. The booklets and the questions they were asked in the PAM appeared to be easy and straightforward to follow, although some comments revealed that the PAM questionnaire was not specific enough to identify their personal abilities and needs. Participants set a range of goals with their stroke nurse. These encompassed exercising, mobilising independently, losing weight, reducing smoking, and improving brain and memory function to increase confidence with socialising. As the free text comments illustrate (Table 4), participants found the goal setting process of particular value. Overall, participants reported that they felt they had been offered appropriate, relevant and timely self-management information and advice during the intervention and several stated this had helped increase their understanding of the effects of their stroke and their confidence to manage their lives (Table 4). Only one participant (SS8) reported that he did not feel that his knowledge, confidence or skills in raising concerns with health professionals had changed over the course of the intervention.

#### Stroke nurses’ perceptions of the intervention

The goal setting process was perceived by stroke nurses as the most valuable component of the intervention and one which had a place in their daily clinical practice. As shown in Table 5, it appeared that it was not only the process of setting goals that made a difference, but the nurses also perceived that the underlying philosophy of a person-centred approach to goal setting achieved through the use of motivational interviewing, as well as the documentation of this, helped to uncover stroke survivors’ personal aspirations and priorities whereas these may not have been revealed otherwise [32]. Goal setting also helped to provide a structure or focus to subsequent visits and self-management discussions, and helped to motivate individual patients and give them a sense of ‘permission’ to begin to take appropriate self-management action. However, it was also acknowledged that the process of ‘goal setting’ required a pre-existing degree of motivation on the individuals’ part; goal setting based interventions may therefore be less successful with less motivated individuals. This corresponds with the findings from the *development* phase, which emphasised the importance of stroke survivors themselves identifying and voicing goals that hold personal or significant meaning.

The PAM was perceived by the nurses as being a less useful component within the tailored self-management action plan (Table 6). In particular, they commented that participants would frequently give a ‘socially desirable’ answer, placing them at the higher end of the self-management spectrum that did not reflect their verbalised (and observed) attitudes or behaviours towards engaging in self-management. The nurses also commented that there was difficulty understanding several questions because of the way they were worded. It was also noted that completing the PAM might be particularly difficult for stroke survivors with communication problems and that more severely impaired stroke survivors may have specific issues of concern not addressed by the PAM.

Despite attempts by the research team to ensure congruence with practice, the intervention as a whole did not appear to fit well into nurses’ daily clinical schedules. The majority of the nurses perceived the self-management approach followed in this intervention as time consuming and increasing their workload (Table 7). The intervention was also viewed by some of the nurses as an adjunct to on-going care, rather an approach that could be readily integrated within their routine practice.

## Discussion

### Feasibility and acceptability of the intervention

This is the first study in the UK to explore the use of ‘patient activation’ to underpin the design and development of a tailored, person-centred stroke self-management support intervention, comprising individualised, systematic assessment of stroke survivors’ needs, expectations and abilities to self-manage and aligning nurses’ provision of support towards these. To our knowledge, it is the first study to involve stroke survivors and stroke nurses in the design and development of a stroke self-management support intervention and to situate the implementation of this within the ‘real world’ community setting, aligning to stroke survivors’ personal needs, abilities, priorities and preferences, and fitting in with nurses’ current roles and workloads.

Many of the individual components of the intervention were well received by stroke survivors and stroke nurses. In particular, the tailored and personalised approach offered by the use of the goal-setting, underpinned by motivational interviewing, was perceived as particularly valuable by both stroke survivors and stroke nurses, and was feasible in the context of nurses’ practice in supporting self-management. The emphasis on the goals being patient-initiated and patient-articulated, being personally meaningful and often outside of the traditional realm of health services, as well as the process of documenting and recording these in an explicit and systematic manner, provided a valuable structure to delivery of self-management support and engaging individuals in meaningful self-management. These elements were seen as different from the nurses’ previous practice. Previous research has identified that patients consider active participation in goal setting and the identification of personally meaningful goals as important following stroke [33, 34]. The mere act of using an explicit method to elicit, document, record and monitor can improve patients’ active participation in the goal setting process [35]; a deeper understanding of this in the context of nurses’ daily practice was developed through the current study. Further work to explore this, and refine the processes of undertaking ‘person-centred goal setting within the context of community-based stroke nurses practice would be valuable.

There was a recognized need for a self-management assessment tool that could be effectively and easily incorporated into nurses’ existing practice, however, the use of the PAM - in its current version – appeared to be a less acceptable component of the intervention. Although the clinical utility of the PAM has been suggested in the literature [20], there remains little understanding of the meaningfulness of the ‘scores’ in relation to actual self-management behavior and action, and little guidance for health professionals on how to use such ‘scores’ to tailor the delivery of self-management support. There is also little understanding of whether the PAM is more applicable to certain long-term conditions than others. The findings from this study suggest that the specific needs of stroke survivors, and indeed subsequent information needed by stroke nurses to inform the delivery of their self-management support, may not be captured and addressed by the current version of the measure. Future work may explore this further and would complement emerging work on outcomes and outcome measures that capture issues relevant to stroke survivors, from a range of stakeholders’ perspectives, including stroke survivors themselves [19, 36].

Despite our attempts to ground the intervention components and delivery within nurses’ routine practice, the intervention was viewed as time consuming, not fitting within the current pattern of scheduled visits and different from what nurses currently do. This mirrors previous research [37] where a main barrier to the implementation of a self-management support intervention was a lack of a shared conceptual understanding of stroke self-management support and ‘person-centered goal setting’ and what this entailed for health professionals within their roles e.g. becoming the role of facilitator and working in partnership with their patients. It appeared that the provision of stroke self-management support was viewed as an adjunct to nurses’ daily practice rather than underpinning it. Although the principles of delivering person-centred care are inherent in the curriculum of practitioners’ training, these can be difficult to carry out in a consistent manner within a time- and resource-squeezed NHS [38]. Further discussions of how stroke self-management and stroke self-management support is conceptualised across nursing and allied health professional circles, adding an interdisciplinary perspective, may help to ensure quality, continuity and consistency of the messages and support used to frame stroke self-management and encourage ‘buy in’ to new approaches to delivering self-management support across teams.

### Limitations

The study findings provide the first step in the development and evaluation of a nurse-led stroke self-management support intervention. It is acknowledged that the views represented here are from a small sample of stroke survivors, the majority of whom were between 1 and 6 months post stroke, male and who did not experience any particularly severe cognitive, communicative and/or visual impairments. Furthermore, participants in the evaluation phase were selected by nurses, which may have introduced a degree of selection bias. The findings from the study sample are, therefore, by no means generalizable and the development of stroke self-management support interventions for people beyond 6–12 months post stroke, for a larger sample of males and females and for those with more severe levels of impairment needs to be explored. Nonetheless, these findings do help to inform the future design and development of research in an area that is likely to grow in the future. It is also acknowledged that the study included a small sample of stroke nurses, based in one NHS Board, and that the intervention period itself was relatively short. It is possible that different views might have been obtained from a larger sample of nurses with differing experiences or from different NHS Boards or had there been a longer intervention period with more time allowed for the provision of self-management support, or further feedback sessions, during the intervention. The individual sessions between the nurses and stroke survivors were not recorded and, therefore, it was not possible to identify whether nurses had adhered to the protocol as planned or whether there were any deviations that could have been further explored in the evaluation focus group. We also did not ask the stroke nurses whether they would continue to use any of the individual intervention components in their daily practice, after the study was completed. These limitations will be addressed in future research.

## Conclusion

This study aimed to develop, implement and qualitatively evaluate a nurse-led stroke self-management support intervention for stroke survivors living at home, from the perspectives of stroke survivors and stroke nurses. Many of the individual components of the intervention were perceived as feasible and acceptable to both stroke survivors and stroke nurses, however, further work is needed to develop and refine the intervention components and address issues related to delivery, implementation, and embedding within nurses’ existing clinical practice.
